# Atraumatic Hepatic Laceration with Hemoperitoneum

**DOI:** 10.3390/diagnostics14182088

**Published:** 2024-09-21

**Authors:** Gaetano Maria Russo, Evangelia Zoi, Imma D’Iglio, Maria Luisa Mangoni di Santo Stefano

**Affiliations:** Ospedali Riuniti Area Nolana, 80035 Napoli, Italy

**Keywords:** liver laceration, hemoperitoneum, atraumatic injury

## Abstract

**Introduction:** A rare case of atraumatic liver laceration associated with hemoperitoneum is presented in a patient with amyloidosis who came to the hospital for abdominal pain. **Case Presentation:** The imaging findings reveal significant hepatomegaly with finely heterogeneous hepatic density and subcapsular hypo-dense streaks in segments VI and VII, likely representing lesions. Post-contrast enhancement shows a punctiform contrast medium extravasation within the subhepatic fluid collection, visible from the arterial phase and intensifying in subsequent study phases. **Discussion:** These imaging findings suggest an atraumatic hepatic laceration, a diagnosis confirmed by the presence of hemoperitoneum distributed bilaterally under the diaphragm, in the paracolic gutters, along the mesentery root, and predominantly in the peri-hepatic region. **Conclusion:** The detailed imaging analysis provided critical insights into the diagnosis and management of this rare clinical presentation.

**Figure 1:**
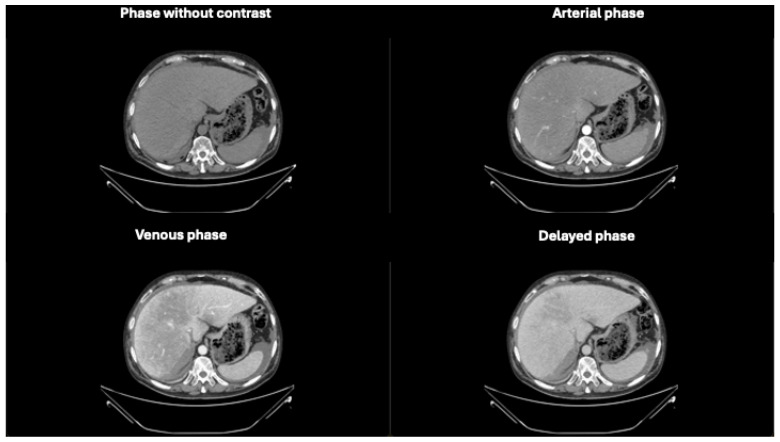
Axial phases without and with contrast CT images demonstrating hepatomegaly with a liver length of approximately 25 cm. The liver parenchyma displays a finely and diffusely heterogeneous density.

**Figure 2:**
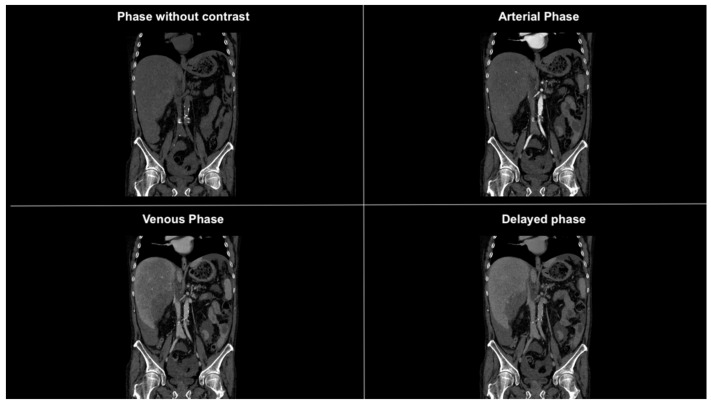
Coronal CT images in different phases showing subdiaphragmatic fluid collection bilaterally, within the paracolic gutters, and prominently around the liver. The fluid collection is suggestive of hemoperitoneum.

**Figure 3:**
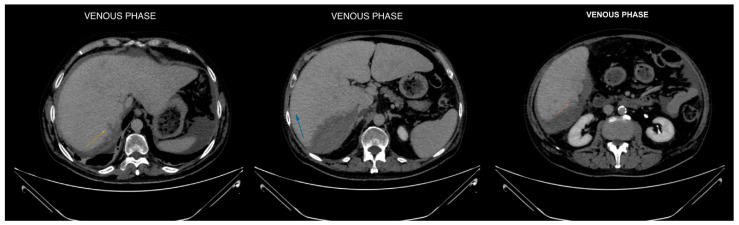
Axial CT images in venous phase focused on liver segments VI and VII, revealing hypodense subcapsular streaks (maximum extension of approximately 1.5 cm). These findings are consistent with hepatic lesions, likely indicative of a laceration [1,2].

**Figure 4:**
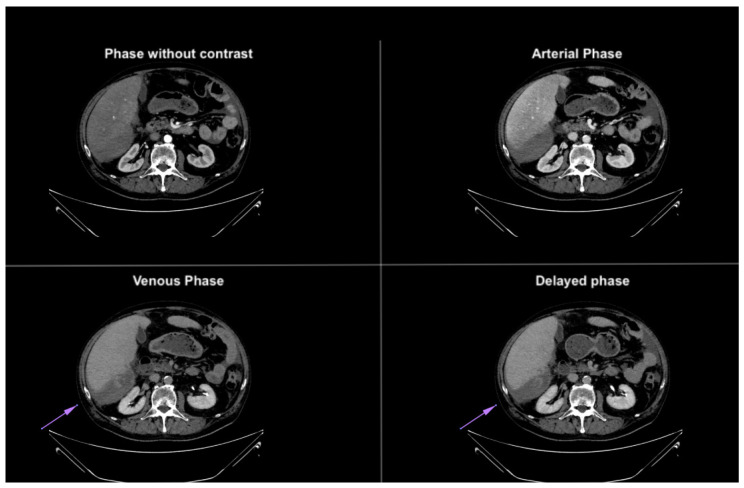
Axial CT images in different phases, highlighting extravasation of contrast medium within the subhepatic fluid collection. The extravasation becomes more prominent in the last phases, confirming the presence of active bleeding. The purple arrow demonstrates blood extravasation in the collection during the venous and delayed phases of the study, not visible in the baseline and arterial phases.

**Figure 5:**
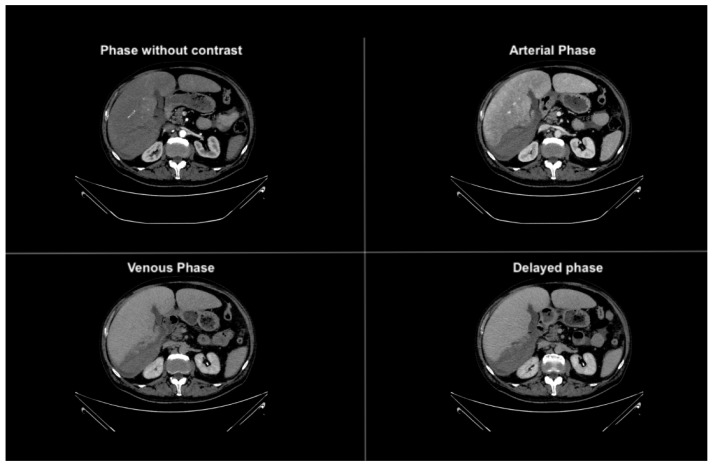
Axial CT images in different phases, highlighting extravasation of contrast medium within the subhepatic fluid collection. In the venous and delayed contrast-enhanced phases, a difference in the density of the perihepatic fluid collection is observed, indicating ongoing active bleeding. The lower border of the liver shows a blurred contour, especially evident along its inferior surface [3].

## References

[1] Bujanda L., Beguiristain A., Alberdi F., Cosme A., Ruíz de la Hermosa J., Gutiérrez-Stampa F., Arenas J.I. (1997). Spontaneous rupture of the liver in amyloidosis. Am. J. Gastroenterol..

[2] Huang B., Gkekas I., Sparellid E. (2019). Amyloidosis and Spontaneous Liver Bleeding: A Case Report and Literature Review. J. Surg..

[3] Leonard-Murali S., Nasser H., Ivanics T., Woodward A. (2019). Spontaneous hepatic rupture due to primary amyloidosis. BMJ Case Rep..

